# Hypothalamic-pituitary-adrenal axis functioning and dysfunctional attitude in depressed patients with and without childhood neglect

**DOI:** 10.1186/1471-244X-14-45

**Published:** 2014-02-18

**Authors:** Hongjun Peng, Ying Long, Jie Li, Yangbo Guo, Huawang Wu, YuLing Yang, Yi Ding, Jianfei He, Yuping Ning

**Affiliations:** 1 Guangzhou Psychiatric Hospital, Affiliated Hospital of Guangzhou Medical University, Guangzhou, China; 2 Guangzhou Civil Affairs Bureau Psychiatric Hospital, Guangzhou, China

**Keywords:** Depression, Childhood neglect, HPA axis functioning, Dysfunctional attitude scale

## Abstract

**Background:**

To date, the relationships between childhood neglect, hypothalamic-pituitary-adrenal (HPA) axis functioning and dysfunctional attitude in depressed patients are still obscure.

**Methods:**

The Childhood Trauma Questionnaire (CTQ) was used to assess childhood emotional neglect and physical neglect. Twenty-eight depressed patients with childhood neglect and 30 depressed patients without childhood neglect from Guangzhou Psychiatric Hospital were compared with 29 age- and gender-matched control subjects without childhood neglect and 22 control subjects with childhood neglect. Cortisol awakening response, the difference between the cortisol concentrations at awakening and 30 minutes later, provided a measure of HPA axis functioning. The Dysfunctional Attitude Scale measured cognitive schema.

**Results:**

HPA axis functioning was significantly increased in depressed patients with childhood neglect compared with depressed patients without childhood neglect (p < 0.001). HPA axis activity in the control group with childhood neglect was significantly higher than in the depressed group without childhood neglect (p < 0.001). Total scores of childhood neglect were positively correlated with HPA axis functioning and dysfunctional attitude scores, but not with severity of depression. We did not find correlations with HPA axis functioning and dysfunctional attitude or with the Hamilton Rating Scale for Depression scores.

**Conclusions:**

Childhood neglect may cause hyperactivity of the HPA axis functioning and dysfunctional attitude, but does not affect depression severity.

## Background

Children with childhood trauma such as abuse, neglect, and loss of young parents are more likely to suffer from depression in adulthood [1-6]. Cognitive vulnerability theory suggests that childhood trauma can lead to adverse cognitive schema, which is the basis of negative automatic thinking in depression [1,7]. Biological vulnerability theory suggests that childhood trauma increases vulnerability to depression through the interaction of genetics and one’s environment [8]. Childhood trauma increases hypothalamic-pituitary-adrenal (HPA) axis activity, potentially mediating environmental risk for developing depression [9]. The current literature indicates HPA axis hyperactivity in patients with depression, and this HPA axis hyperactivity is thought to be the outcome of childhood trauma and a predisposing factor to depression rather than the outcome of depression [1]. However, HPA axis functioning hypoactivity has also been found in patients with depression [10]. For example, one study reported that the cortisol awakening response was decreased after an early loss experience [11]. However, another study found that parental loss was associated with increased cortisol awakening responses [12].

Childhood neglect has the highest prevalence among types of childhood trauma in the United States [13,14]; however, studies on childhood neglect have found inconsistent results regarding the impact of childhood neglect on cortisol levels and HPA axis functioning [12]. A study using the dexamethasone/corticotropin-releasing hormone test to measure HPA axis functioning found no significant differences in HPA axis functioning between depressed patients with high levels of childhood emotional neglect and healthy controls, but found significantly increased HPA axis functioning in depressed patients with low levels of childhood emotional neglect [15]. Possible explanations for this finding may be that childhood neglect did not change the activity of the glucocorticoid receptors (GR), or that increased GR activity was offset by the hypoactivity caused by genetic factors. Another study found that cortisol levels were significantly different between depressed patients with and without childhood emotional neglect, but there was no significant difference among different types of childhood trauma classified by the Childhood Trauma Questionnaire (CTQ) subscale scores [16]. The authors of that study proposed that emotional neglect was one of the most common types of childhood trauma, and that the lasting effect of chronic psychological stress on the HPA axis resulted in neurobiological changes [17]. In addition, Beck suggested that childhood adversities might distort cognition about oneself and the world, and that depressed patients with childhood neglect often show dysfunctional attitudes, or a somewhat distorted cognitive schema [18]. Some studies have suggested that a dysfunctional attitude is related to severity of depression, while others have proposed that it is a trait resulting from childhood trauma, and is not related to severity of depression [19,20]. At present, the relationships among childhood neglect, HPA axis functioning and dysfunctional attitude remain obscure. Therefore, in this study, we attempted to explore the impact of childhood neglect on HPA axis functioning, dysfunctional attitude and the severity of depression.

## Methods

### Subjects

We recruited 124 depressed patients between the ages of 18 and 45 years from the inpatient and outpatient units of Guangzhou Psychiatric Hospital, Affiliated Hospital of Guangzhou Medical University. The structured clinical interview (SCID) for DSM-IV diagnostic criteria was used to assess the presence or absence of major depressive disorder (MDD) [21]. Any patient with other psychiatric axis-I or axis-II disorders, any other neurological disorder, any substance use within the past 6 months, electroconvulsive therapy, and any other clinically relevant abnormalities in their medical history or laboratory examinations was excluded. A 17-item Hamilton Depressive Rating Scale (HAMD) was used to evaluate the severity of depression [22]. The CTQ [23-25] was used to evaluate childhood trauma. Childhood neglect included emotional and physical neglect and the co-occurrence of both of them according to the subscale scores of the CTQ. The cutoff score for moderate-severe exposure was set at ≥15 for emotional neglect, ≥10 for physical neglect, and ≥20 for the coexistence of both of them. We excluded any other type of child trauma (any other CTQ subscale scores were required to be lower than 8) [23,26]. According to the cutoff values, after excluding subjects whose salivary samples were polluted by minor bleeding in the oral cavity, or samples that were collected after having breakfast, drinking something or brushing teeth during the first half hour after awakening, we had a total of 28 patients with childhood neglect (mean age, 28.87 ± 6.28 years; mean disease course, 21.68 ± 22.12 months; male/female, 15/13) and 30 patients without child neglect (excluding any other type of trauma) (mean age, 28.37 ± 8.27 years; mean disease course, 25.48 ± 19.06 months; male/female, 16/14). There were no significant between-group differences in age, gender, disease course, severity of depression and treatment process. In addition, 29 sex- and age-matched healthy controls without childhood neglect (mean age, 27.87 ± 4.28 years; male/female, 15/14) and 22 healthy controls with childhood neglect (mean age, 28.37 ± 5.28 years; male/female, 12/10) were recruited from the local community. Before enrollment, all subjects including patients and controls were fully informed about the study and written informed consent was obtained. These studies were performed according to the Declaration of Helsinki and approved by the Guangzhou Psychiatric Hospital ethics committee.

### Childhood trauma questionnaire

The Childhood Trauma Questionnaire (CTQ) was used to examine childhood abuse and neglect. This is a reliable and valid self-reporting questionnaire with 28 items used to evaluate childhood trauma severity in five aspects including childhood physical abuse, emotional abuse, sexual abuse, emotional neglect and physical neglect. Each subscale is measured with five items and rated on a five-point Likert scale [24,25].

### HPA axis functioning evaluation

The salivary cortisol awakening response (CAR) [27] was used to evaluate HPA axis functioning [16,27-29]. Salivary cortisol samples were obtained at awakening and 30-min after awakening over two consecutive days, and HPA axis functioning was defined as the difference between cortisol concentrations at 30 min after awakening and at awakening. We used the mean value of the consecutive two days as the index of HPA axis functioning. Previous studies have showed that the net cortisol awakening increase between time of awakening and 30-min after awakening is sensitive to group differences [30,31].

### Dysfunctional attitude evaluation

The Dysfunctional Attitudes Scale (DAS) [32] is a 40-item questionnaire designed to measure cognitive vulnerability to depression, which may reflect the impact of early negative events on one’s cognition about self and the world. Higher scores reflect more dysfunctional attitudes [20].

### Social support rating scale

The Social Support Rating Scale (SSRS) [33] is a 10-item self-report questionnaire, designed by Xiao during 1986-1993 and widely used in China, that includes measurements of subjective support, objective support, and utilization of social services.

### Salivary sample collection and cortisol assay

We assessed early morning salivary cortisol levels at home on two consecutive work days (excluding Monday) with the Salivette sampling device (Salimetrics). The saliva samples at 0 and 30 minutes after awakening were collected on each day, and subjects were instructed not to brush their teeth, take food or smoke before completing saliva sampling. Samples were stored in the subject’s home freezer and the next day were sent to the laboratory and frozen at -20°C until assayed. On the day of the assay, the salivettes were centrifuged for 10 min, at 3000 rpm at 4°C. All samples were assayed in duplicate using a high sensitivity salivary cortisol enzyme immunoassay kit (Salimetrics, State College, PA) for quantitative measurement of salivary cortisol [34]. Samples from each participant were assayed in the same batch. The interassay variability was 8.1%; the intra-assay variation was 8.5%.

### Statistical analysis

Two-way Analyses of variance (ANOVA) was used to compare differences of HPA axis functioning, dysfunctional attitude and social support scores among the four groups: depressives with childhood neglect, depressives without childhood neglect, healthy controls with childhood neglect and healthy controls without childhood neglect. Post-hoc testing was used to compare differences of above-mentioned indexes between any two groups. Relationships among childhood neglect, HPA axis functioning, dysfunctional attitude and social support scores were assessed by means of Pearson correlations. All analyses were conducted using SPSS 17.0 for Windows.

## Results

### The differences of HPA axis functioning, dysfunctional attitude and Social Support Rating Scale scores among depressed groups with child neglect and without childhood neglect and healthy control groups

As shown in Table 1, there were significant differences of HPA axis functioning, dysfunctional attitude scores and Social Support Rating Scale scores among the healthy control groups and the depressed patients with childhood neglect and without childhood neglect.

**Table 1:** Differences in HPA axis functioning, DAS, and SSRS between depressed and control groups with and without childhood neglect

	**CWOCN (n = 29)**	**DWCN (n = 28)**	**DWOCN (n = 30)**	**CWCN (n = 22)**	**F**	**p**
HPA axis functioning (nmol/l)	6.82 ± 2.81	8.76 ± 2.43	4.84 ± 2.73	7.68 ± 1.66	5.92	0.001
DAS	114.21 ± 21.38	159.72 ± 31.28	139.17 ± 28.87	142.20 ± 26.3	25.42	0.000
SSRS	40.18 ± 6.33	31.72 ± 6.94	30.36 ± 6.72	41.23 ± 8.52	9.01	0.000

*CWOCN*, Healthy control without childhood neglect; *DWCN*, Depression with childhood neglect; *DWOCN*, Depression without childhood neglect; *CWCN*, Healthy control with childhood neglect; *HPA axis functioning*, Cortisol concentrations at 30 minutes after awakening -cortisol concentrations at awakening; *SSRS*, Social Support Rating Scale; p < 0.001 represent significant differences statistically.

### Post-hoc testing in HPA axis functioning and dysfunctional attitude scores among depressed group with childhood neglect and without childhood neglect and healthy control groups

As shown in Table 2, Post-hoc testing indicated that HPA axis functioning was significantly increased in depressed patients with childhood neglect compared with depressed patients without childhood neglect (p < 0.001); the HPA axis functioning in the healthy controls with childhood neglect was significantly higher than that in the depressed patients without childhood neglect (p < 0.01). We did not find significant differences in dysfunctional attitude scores between depressed patients without childhood neglect and healthy controls with childhood neglect (p > 0.05).

**Table 2:** Differences in HPA axis functioning, DAS scores between any two groups of healthy control groups, depressed groups with and without childhood neglect

**Comparison between the two groups**		**HPA axis functioning (nmol/l)**	**p**	**DAS**	**p**
CWOCN		6.82 ± 2.81		114.21 ± 21.38	
	DWCN	8.76 ± 2.43	0.067	159.72 ± 31.28	0.000
	DWOCN	4.84 ± 2.73	0.048	139.17 ± 28.87	0.000
	CWCN	7.68 ± 1.66	0.29	142.20 ± 26.3	0.000
DWCN		8.76 ± 2.43		159.72 ± 31.28	
	DWOCN	4.84 ± 2.73	0.000	139.17 ± 28.87	0.000
	CWCN	7.68 ± 1.66	0.59	142.20 ± 26.3	0.006
DWOCN		4.84 ± 2.73		139.17 ± 28.87	
	CWCN	7.68 ± 1.66	0.007	142.20 ± 26.3	0.881

*CWOCN*, Healthy control without childhood neglect; *DWCN*, Depression with childhood neglect; *DWOCN*, Depression without childhood neglect; *CWCN*, Healthy control with childhood neglect; *HPA axis functioning*, Cortisol concentrations at 30 minutes after awakening -cortisol concentrations at awakening; p < 0.05 represent significant differences statistically.

### The correlations among the total scores of childhood neglect, HPA axis functioning, HAMD, DAS, and SSRS scores

**Figure 1:**
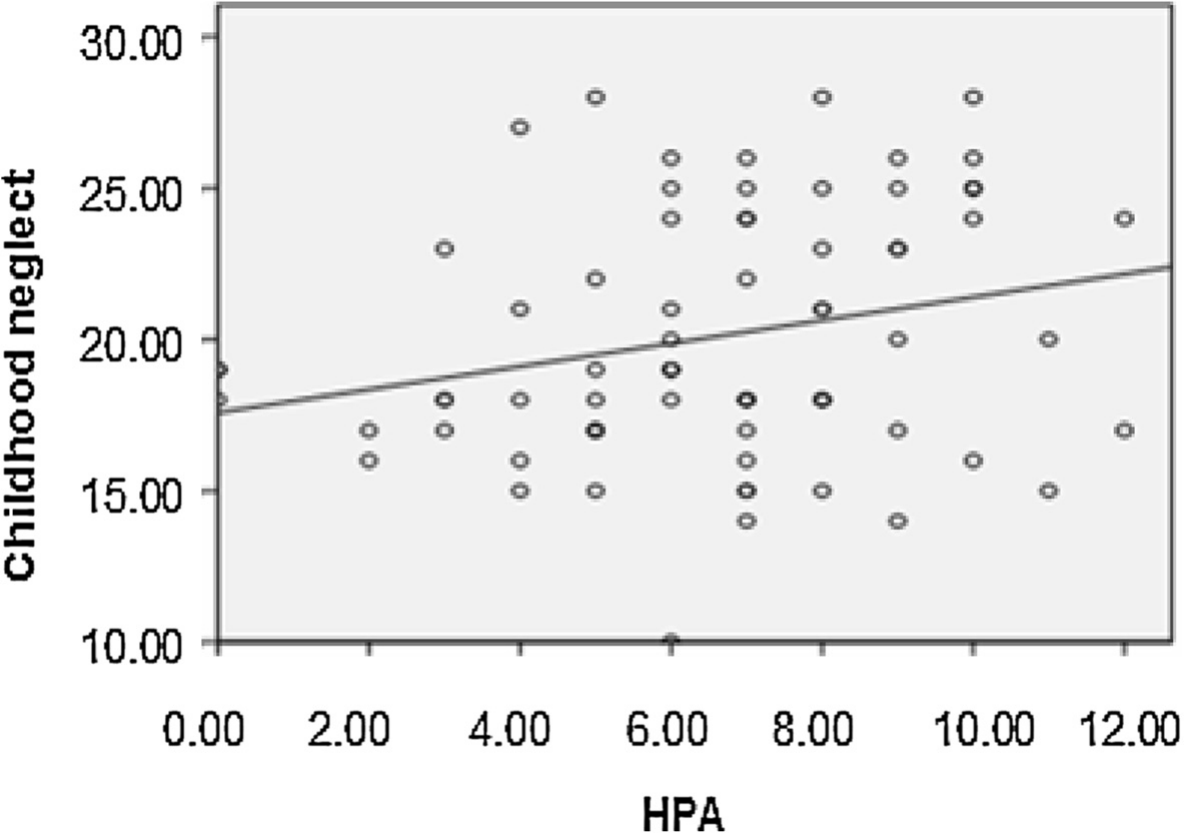
The correlation between childhood neglect and HPA axis functioning in all subjects.

**Figure 2:**
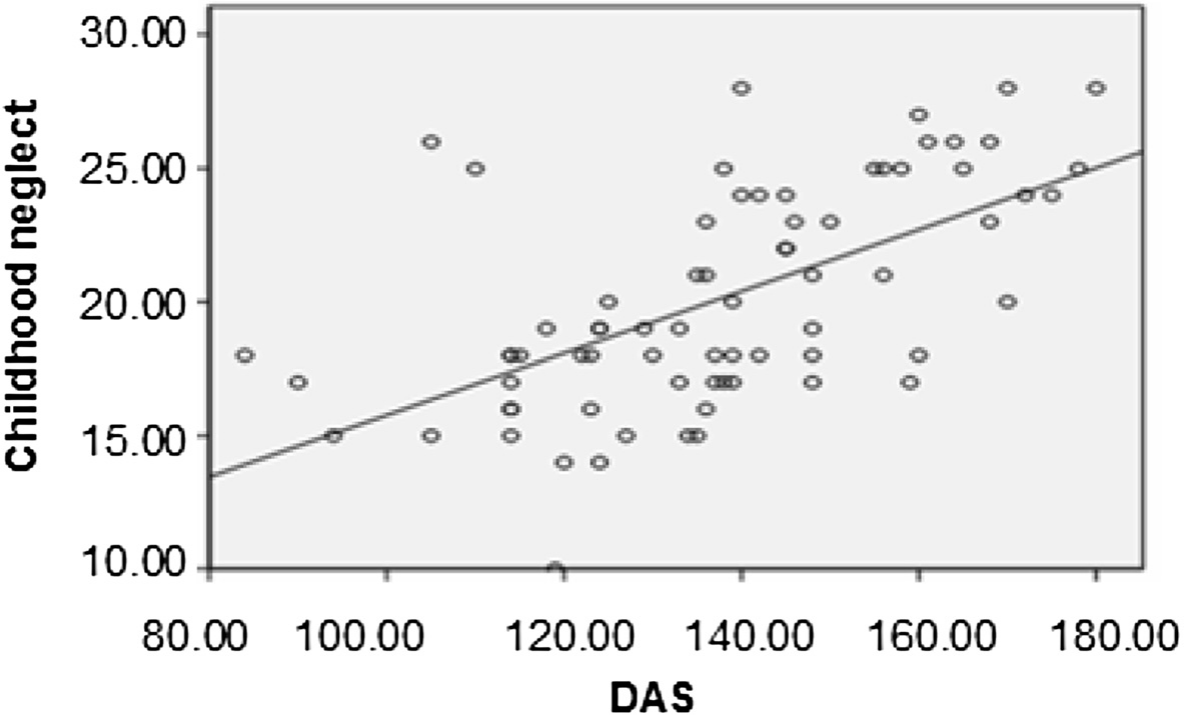
The correlation between childhood neglect and dysfunctional attitude scales (DAS) scores in all subjects.

**Figure 3:**
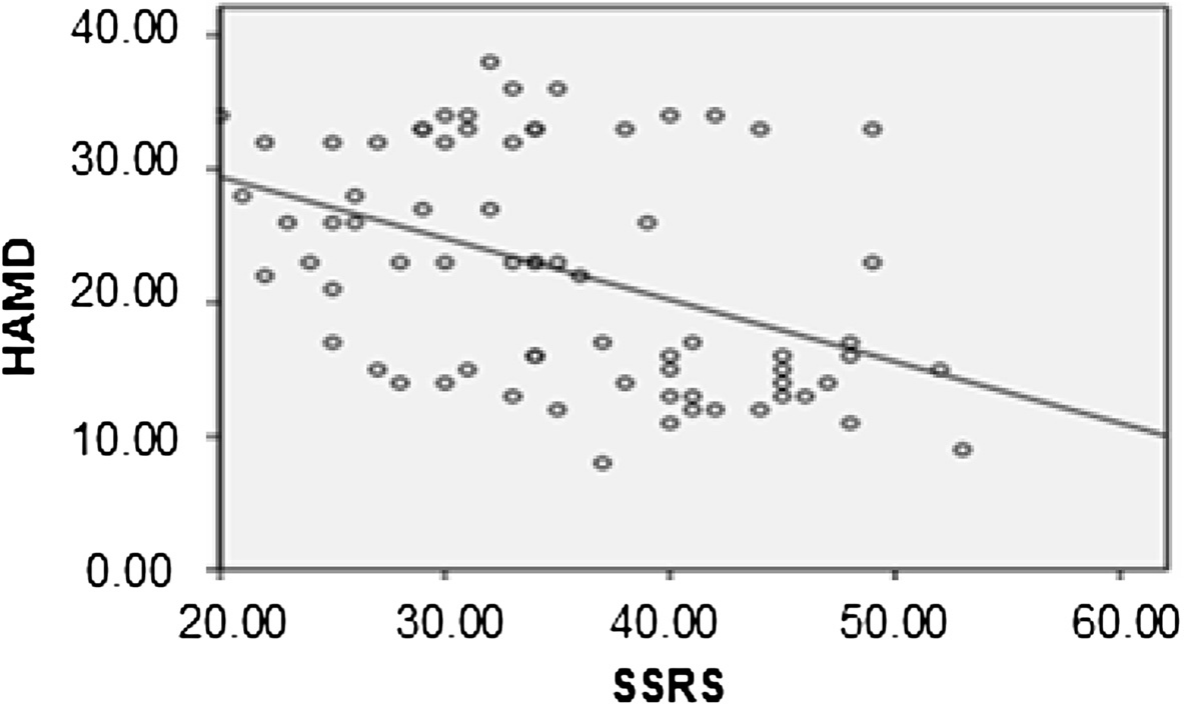
The correlation between HPA axis functioning and social support rating scale scores (SSRS).

As shown in Figure 1 and Figure 2, the total scores of child neglect in all subjects were significantly positively related to HPA axis functioning (r = 0.261, p = 0.032) and DAS scores (r = 0.587, p = 0.000), but not related to HAMD scores (r = 0.131, p = 0.310) (not shown). We also did not find correlations between HPA axis functioning and DAS scores (r = 0.089, p = 0.460) or HAMD scores (r = -0.102, p = 0.401). As shown in Figure 3, we found that the severity of depression was negatively related to social support scores (r = -0.442; p = 0.000).

## Discussion

Previous studies have suggested that childhood neglect could be a predictor of psychopathology, monoamine system dysfunction, and increased cerebrospinal fluid corticotropin-releasing hormone levels in adulthood, when compared with other forms of childhood trauma [16,35,36]. In our study we focused predominantly on the impact of childhood neglect on HPA axis functioning, and Dysfunctional Attitude Scale (DAS) scores. Analysis of variance showed that there were significant differences in HPA axis functioning, DAS scores and Social Support Rating Scale (SSRS) scores among the four groups: depressed patients with childhood neglect, depressed patients without childhood neglect, healthy controls with childhood neglect and healthy controls without childhood neglect. Post hoc testing showed that HPA axis function activity was significantly higher in depressed patients with childhood neglect than in depressed patients without childhood neglect. This finding is consistent with previous studies and suggests that childhood neglect is related to hyperactivity of the HPA axis functioning. Animal experiments have demonstrated that when newborn rats and non-human mammals were separated from their mothers for a period of time, HPA axis activity increased and continued to increase into adulthood [6]. Clinical studies have shown that HPA axis activity increased in men with childhood trauma [9] and that women who suffered from childhood trauma showed adrenocorticotropic hormone hyperactivity and increased heart rates as adults after performing the Trier Social Stress Test, even if they did not suffer from depression in adulthood [37]. In addition, we found that HPA axis activity was higher in the healthy control group with childhood neglect than in depressed patients without childhood neglect. This finding suggests that HPA axis activity was not always higher in the depressed patients than in healthy controls, and that childhood neglect might play an important role in the hyperactivity of the HPA axis functioning [9,37].

The DAS is a 40-item questionnaire designed to measure cognitive vulnerability for depression. Studies have reported that DAS scores were positively related to the Hamilton Rating Scale for Depression (HAMD) scores [32], while others have thought that dysfunctional attitude was not state-dependent or symptom-dependent, but a trait associated with childhood trauma [18,38,39]. Our results showed that DAS scores were not related to HAMD scores, but were positively related to childhood neglect scores. This supports the hypothesis that dysfunctional attitude is a trait resulting from childhood neglect. In our study we found that DAS scores were lowest in the healthy control group without childhood neglect and highest in depressed patients with childhood neglect. This finding provides further support for the hypothesis that childhood neglect plays an important role in the formation of dysfunctional attitude. Beck suggested that adversities that occur in childhood might distort cognition about oneself and the world, and that dysfunctional attitudes might underlie the negative automatic thoughts of depression [19,20].

Our results demonstrated that HPA axis functioning was related to childhood neglect but not to the severity of depression, which is consistent with findings from previous studies. Over the last 40 years, several studies have proposed that HPA axis hyperactivity is not a biological outcome of depression, but a predisposing factor that often results from childhood trauma [40,41]. Therefore, childhood neglect has been related to HPA axis functioning and HPA axis hyperactivity might be one of the risk factors for depression.

In addition, we did not find a correlation between childhood neglect and HAMD scores, but we found that SSRS scores were negatively related to HAMD scores. This might suggest that social support was helpful in alleviating the symptoms of depression. In our study, we found that the healthy control groups had higher SSRS scores than the depressed groups, which implies that social support might prevent depression.

Limitations: Our sample was relatively small. We used saliva cortisol awakening response as an index of HPA axis functioning, selecting only two time points (0 min, 30 min after awakening) on 2 consecutive days to collect the saliva sample. Though one study has reported this method to be sensitive and reliable [42], further research should duplicate the results of the current study in a larger sample and with an optimized method of sampling over five time points (0 min, 15 min, 30 min, 45 min, 60 min after awakening) for cortisol collection. There are several potential unmeasured confounders known to be associated with cortisol collection, for example, compliance and sleep quality. In addition, the CTQ was used to measure childhood trauma; the self-report nature of this measure may have caused recall bias. We tried to overcome this problem by obtaining information about the subjects’ early life experiences from their first relatives. Finally, we suggest that more advanced statistical methods and a more powerful p-value correction should be performed in future research of this topic.

## Conclusions

Our results demonstrated that childhood neglect was positively related to HPA axis functioning and dysfunctional attitude, but not to the severity of depression. Our results might suggest that childhood neglect could lead to one’s physical and psychological changes.

## Competing interests

The authors declare that they have no competing interests.

## Authors’ contributions

Authors HP and YN designed the study and developed the protocols. YN and JL are tutors of HP. Authors HP, YL, JL, YG, and HW carried out literature searches and analyses. Authors YY, YD, JH and HP performed statistical analyses and prepared the first draft of the manuscript. All authors read and approved the final manuscript.

## Pre-publication history

The pre-publication history for this paper can be accessed here:

http://www.biomedcentral.com/1471-244X/14/45/prepub
